# Prevalence and characterization of antimicrobial resistance among gram-negative bacteria isolated from febrile hospitalized patients in central Ethiopia

**DOI:** 10.1186/s13756-022-01053-7

**Published:** 2022-01-15

**Authors:** Tafese Beyene Tufa, Colin R. Mackenzie, Hans Martin Orth, Tobias Wienemann, Tamara Nordmann, Sileshi Abdissa, Zewdu Hurissa, Andreas Schönfeld, Matthias Bosselmann, Dieter Häussinger, Klaus Pfeffer, Tom Luedde, Andre Fuchs, Torsten Feldt

**Affiliations:** 1 College of Health Sciences, Arsi University, P.O. Box 04, Asella, Ethiopia; 2 Department of Gastroenterology, Hepatology and Infectious Diseases, University Hospital Duesseldorf, Medical Faculty of Heinrich Heine University, Moorenstr. 5, 40225 Duesseldorf, Germany; 3 Present Address: Hirsch Institute of Tropical Medicine, P.O. Box 04, Asella, Ethiopia; 4 Institute of Medical Microbiology and Hospital Hygiene, University Hospital Duesseldorf, Medical Faculty, Heinrich Heine University, Universitätsstr. 1, 40225 Duesseldorf, Germany; 5 Division Tropical Medicine, Department of Medicine, University Medical Center Hamburg-Eppendorf, Bernhard-Nacht-Straße 74, 20359 Hamburg, Germany; 6 Department of Infectious Diseases, Essen University Hospital, Essen, Germany; 7 Internal Medicine III – Gastroenterology and Infectious Diseases, University Hospital of Augsburg, Stenglinstr. 2, 86156 Augsburg, Germany

**Keywords:** Antimicrobial resistance, CTX-M-1, TEM, NDM-1, ESBL, Carbapenemase, Sub-Saharan Africa

## Abstract

**Background:**

Infectious diseases are among the leading causes of death in many low-income countries, such as Ethiopia. Without reliable local data concerning causative pathogens and antimicrobial resistance, empiric treatment is suboptimal. The objective of this study was to characterize gram-negative bacteria (GNB) as pathogens and their resistance pattern in hospitalized patients with infections in central Ethiopia.

**Methods:**

Patients ≥ 1 year of age with fever admitted to the Asella Referral and Teaching Hospital from April 2016 to June 2018 were included. Blood and other appropriate clinical specimens were collected and cultured on appropriate media. Antibiotic susceptibility testing (AST) was performed using the Kirby–Bauer method and VITEK® 2. Species identification and detection of resistance genes were conducted using MALDI-ToF MS (VITEK® MS) and PCR, respectively.

**Results:**

Among the 684 study participants, 54.2% were male, and the median age was 22.0 (IQR: 14–35) years. Blood cultures were positive in 5.4% (n = 37) of cases. Among other clinical samples, 60.6% (20/33), 20.8% (5/24), and 37.5% (3/8) of swabs/pus, urine and other body fluid cultures, respectively, were positive. Among 66 pathogenic isolates, 57.6% (n = 38) were GNB, 39.4% (n = 26) were gram-positive, and 3.0% (n = 2) were *Candida* species. Among the isolated GNB, 42.1% (16/38) were *Escherichia coli*, 23.7% (9/38) *Klebsiella pneumoniae* and 10.5% (4/38) *Pseudomonas aeruginosa*.

In total, 27/38 gram-negative isolates were available for further analysis. Resistance rates were as follows: ampicillin/sulbactam, 92.6% (n = 25); cefotaxime, 88.9% (n = 24); ceftazidime, 74.1% (n = 20); cefepime, 74.1% (n = 20); gentamicin, 55.6% (n = 15); piperacillin/tazobactam, 48.1% (n = 13); meropenem, 7.4% (n = 2); and amikacin, 3.7% (n = 1). The *bla*_NDM-1_ gene was detected in one *K. pneumoniae* and one *Acinetobacter baumannii* isolate, which carried an additional *bla*_OXA-51_ gene. The ESBL enzymes were detected in 81.5% (n = 22) of isolates as follows: TEM, 77.2% (n = 17); CTX-M-1 group, 68.2% (n = 15); SHV group, 27.3% (n = 6); and CTX-M-9 group, 9.1% (n = 2). Based on the in vitro antimicrobial susceptibility results, empiric treatment initiated in 13 of 18 (72.2%) patients was likely ineffective.

**Conclusion:**

We report a high prevalence of ESBL-producing bacteria (81.5%) and carbapenem resistance (7.4%), with more than half of GNB carrying two or more ESBL enzymes resulting in suboptimal empiric antibiotic therapy. These findings indicate a need for local and national antimicrobial resistance surveillance and the strengthening of antimicrobial stewardship programs.

## Introduction

Infectious diseases are among the leading causes of morbidity and mortality in many low-income countries, such as Ethiopia [1]. Bacterial sepsis is a severe complication of different infections caused by gram-negative bacteria (GNB), leading to high rates of mortality. Pneumonia, bacteremia, urinary tract infection, intra-abdominal infection and skin and soft tissue infection are the major GNB sources of infections [2].

The emerging multidrug resistance (MDR) in GNB has become a serious public health problem worldwide [3]. A major driver of resistance in GNB is the horizontal transfer of mobile genetic elements carrying genes for extended-spectrum β-lactamases (ESBL) and/or carbapenemases [4]. These enzymes hydrolyze penicillins, third-generation cephalosporins (3GCs) and carbapenems. The most common ESBLs are cefotaximase (CTX-M-type), temoniera (TEM), and sulfhydryl reagent variable (SHV). Furthermore, the most prevalent carbapenemases are oxacillinases (OXA), namely, OXA-23, OXA-24/40, OXA-48, OXA-51, OXA-58, OXA-143, and OXA-235; *Klebsiella pneumoniae* carbapenemase (KPC); metallo-beta-lactamases (MBL), such as the New Delhi metallo-β-lactamase (NDM); imipenemase (IMP); and Verona imipenemase (VIM). These enzymes confer resistance to cephalosporins and carbapenems [5, 6].

Despite evidence for high rates of 3GC resistance among gram-negative isolates in many African countries, 3GCs are among the most commonly used antibiotics for the initial empiric treatment of severe infections and sepsis in sub-Saharan Africa (SSA). Data to estimate the mortality associated with antimicrobial resistance (AMR) in SSA are limited [7]. Implementation of antimicrobial stewardship (AMS) programs, improvement of antimicrobial susceptibility testing (AST) capacity, and provision of local and national AMR data for common bacterial pathogens should be given attention in these regions. Continued extensive empiric use of 3GC in the absence of AST might lead to a further decrease in antimicrobial activity and therefore increase the burden on the resource-limited health care systems of the countries affected [8]. This development can also be observed locally, and previous investigations at the study site show that the effectiveness of the limited choice of available antimicrobials is diminished due to the high rate of ESBL-producing GNB [9]. Due to the increase in ESBL-producing GNB-associated infections that are difficult or impossible to treat under local circumstances, there is a growing need for strategies to improve the prudent use of antibiotics in this country [10, 11].

The lack of systematically acquired local data concerning both the causative organisms and common resistance patterns likely results in ineffective empiric treatment and an unfavorable clinical outcome. Therefore, we assessed the rate and extent of drug resistance among GNB isolated from hospitalized patients with infectious diseases at a tertiary hospital in central Ethiopia and characterized the AMR genes.

## Materials and methods

### Study design and inclusion

This prospective operational research was conducted from April 2016 to June 2018 at the Asella Referral and Teaching Hospital (ARTH), a tertiary hospital in the town of Asella, located in the central part of Ethiopia. The bacterial cultures were performed in Ethiopia, and secondary and molecular biological investigations were performed in Germany.

**Fig. 1:**
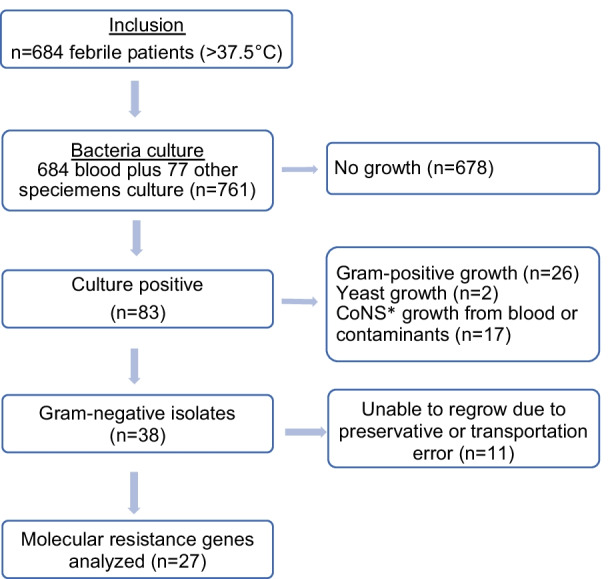
Flow diagram of participants’ inclusion. *CoNS growth was only considered in blood samples and interpreted as contaminants since we only used one set of blood culture bottles

During the study period, all patients ≥ 1 year of age presenting for treatment at ARTH with fever defined as body temperature > 37.5 °C according to tympanic measurement were offered inclusion in the study (Fig. 1). The eligible patients who fulfilled the inclusion criteria were identified by a trained study team. After written informed consent was obtained from the patients or legal guardians, blood cultures (BCs) were drawn from all participants. Previously initiated antibiotic treatment was not an exclusion criterion. In addition to BC, appropriate clinical samples according to the patient’s symptoms and the treating physician’s decision were collected for further microbiological investigation, according to local standard operating procedures (SOPs) and national treatment guidelines. Sociodemographic and clinical data were collected by using a standardized questionnaire.

### Ethical approval and consent to participate

The appropriate ethical review boards of Arsi University (reference number A/U/H/S/C/120/6443/2017), the Oromia Regional Health Bureau (reference number BEFO/AHBTFH/1-8/2017), and Düsseldorf University Hospital (reference number 5729) approved the study. Ethical clearance for sample transportation between Ethiopia and Germany was obtained from the National Ethical Review Board of the Ministry of Science and Technology (reference number 310/204/2017). Before inclusion in the study, written informed consent to participate in the study was obtained from each participant or, in the case of minors, from their legal guardians.

### Blood culture

Approximately 5 ml of blood from each child and 10–20 ml from each adult participant was collected and inoculated into an aerobic blood culture bottle. For the first 200 participants, we used in-house-produced blood culture bottles, and for the remaining participants, we used commercially available blood culture bottles (BacT/ALERT culture media bottles, bioMérieux, Marcy-l’Étoile, France). In both cases, we used the same incubator and did not find a significant difference in the positivity rates except for slightly more contaminants when using the in-house-produced blood culture bottles. The cultures were incubated at 37 °C for a maximum period of five days. After 24 h and at the end of the incubation period, Gram staining and subculturing on blood, MacConkey, and chocolate agar in a candle jar or a 5% CO_2_-enriched atmosphere were performed. Biochemical identification tests were subsequently performed based on the gram staining results. For GNB identification, the biochemical identification tests performed were oxidase, lactose, indole, mannitol, urease, triple sugar iron, citrate, and lysine decarboxylase, whereas for gram-positive bacteria, catalase, coagulase and hemolysis tests were used. Only one aerobic culture was performed per patient for in-house blood culture bottles, and a single pair (one aerobic and one anaerobic culture media bottle) was performed for BacT/ALERT blood culture bottles. Regular quality controls for sensitivity were performed by incubation of ATCC 25922 (*Escherichia [E.] coli*), ATCC 700603 (*Klebsiella [K.] pneumoniae*), ATCC 27853 (*Pseudomonas [P.] aeruginosa*), ATCC 747 (*Acinetobacter [A.] baumannii*) and ATCC 25923 (*Staphylococcus [S.] aureus*).

### Urine culture

In participants with possible urinary tract infection, mid-stream urine specimens were collected using sterile urine cups, and 1 μL of urine was inoculated onto blood and MacConkey agar. We used a semiquantitative urine culture method to interpret the results for female participants. Hence, after 24 h of incubation at 37 °C, bacterial colonies were further processed if the colony count was ≥ 10^5^ CFU/mL.

### Swabs and other body fluid culture

If the patient had abscesses or any wounds, swabs or aspirates of other body fluids were collected using appropriate sterile sampling devices (swabs, sterile syringes, etc.) and cultivated on blood and chocolate agar in a 5% CO_2_-enriched atmosphere. The isolates were further processed following the local laboratory’s SOPs.

### Antimicrobial susceptibility testing

Following the identification of GNB, Kirby–Bauer AST was performed after cultivation on Mueller–Hinton agar. The procedure and interpretation followed the European Committee on Antimicrobial Susceptibility Testing (EUCAST) version 7.1 recommendations. The microbiological results were reported to the treating physicians at ARTH as soon as possible to ensure appropriate patient care.

### Confirmation of species identification and antimicrobial susceptibility test

Bacterial isolates were preserved at −80 °C in Microbank® vials (Pro-Lab Diagnostics Inc., Toronto, Canada). Subsequently, samples of all isolates were exported to Germany for confirmation of species identification and AST as well as molecular biological analysis of resistance mechanisms. Species identification was confirmed by using matrix-assisted laser desorption/ionization-time of flight (MALDI-TOF) mass spectrometry (Vitek® MS, bioMérieu), and the local AST result was confirmed by VITEK® 2 (bioMérieux). There were no significant discrepancies between the Kirby–Bauer and VITEK AST results. However, some discrepancies between the species identification according to the biochemical tests performed in Ethiopia and the MALDI-TOF results occurred. For instance, 81.5% (22/27) of GNB isolates were correctly identified by biochemical tests performed in Ethiopia compared with the MALDI-TOF results.

### ESBL and carbapenemase gene detection

After identification and AST, GNB DNA was extracted by suspending a pure colony grown on MacConkey agar in 200 μL of Tris–EDTA pH 8.0 buffer. The suspension was then heated at 95 °C for 20 min, followed by centrifugation at 10,000 rpm for 10 min. Then, 150 µl of the supernatant was transferred into a new tube and stored at −20 °C until PCR testing could be performed.

All available GNB isolates were investigated by PCR (Fig. 1). We used the PCR protocols described by Strauß et al. for identification of the β-lactamase *bla*_CTX-M_, *bla*_SHV_ and *bla*_TEM_ genes [12] for all available GNB isolates and an in-house PCR protocol established for the detection of the carbapenemase genes *bal*_IMP-1_, *bal*_VIM-1_, *bal*_VIM-2_, *bla*_GIM-1_, *bla*_OXA-23_, *bla*_OXA-24/40_, *bla*_OXA-58_, *bla*_NDM-1_ and *bla*_OXA-51_, as described by Wendel et al. [13, 14] (Table 1) for strains that were resistant to imipenem/meropenem according to the AST results.

**Table 1:** Oligonucleotide sequences of the primer pairs used for molecular resistance gene detection

Primer	Sequence (5′–3′)	Amplicon size (bp)	References
*bla*_SHV_ (F)	AGCCGCTTGAGCAAATTAAAC	786	[12]
*bla*_SHV_ (R)	GTTGCCAGTGCTCGATCAGC		
*bla*_TEM_ (F)	CATTTCCGTGTCGCCCTTATTC	846	[12]
*bla*_TEM_ (R)	CCAATGCTTAATCAGTGAGGC		
*bla*_CTX-M-1_ (F)	CGTCACGCTGTTGTTAGGAA	781	[12]
*bla*_CTX-M-1_ (R)	ACGGCTTTCTGCCTTAGGTT		
*bla*_CTX-M-2_ (F)	CTCAGAGCATTCGCCGCTCA	843	[12]
*bla*_CTX-M-2_ (R)	CCGCCGCAGCCAGAATATCC		
*bla*_CTX-M-9_ (F)	GCGCATGGTGACAAAGAGAGTGCAA	876	[12]
*bla*_CTX-M-9_ (R)	GTTACAGCCCTTCGGCGATGATTC		
*bla*_NDM-1_ (F)	CGG CAT CACCGA GAT TGC	732	[13]
*bla*_NDM-1_ (R)	CACCGA CAT CGC TTTTGGT		
*bla*_OXA-51_ (F)	TGT CTAAGGAAGTGAAGCGTG	112	[14]
*bla*_OXA-51_ (R)	AACTGTGCCTCTTGCTGAG		

*F* forward, *R* reverse, *bp* base pairs

### Data analysis

Data were entered and analyzed using IBM SPSS Statistics for Windows version 25 (IBM Corp., Armonk, NY, USA). Fisher’s exact and Pearson’s chi-squared tests were used for the analysis of quantitative variables, and the mean, median and standard deviation were used for continuous variables. Differences were considered statistically significant at *p* < 0.05.

#### Quality control

For quality control, the control strains listed in Table 2 were used during the resistance gene analysis.

**Table 2:** Control strains used for resistance gene analysis

Resistance gene	Base pairs	Bacterial species	ATCC number
SHV group	780	*Klebsiella pneumoniae*	NRZ-02718
TEM group	860	*Klebsiella oxytoca*	NRZ 09574
CTX-M-1 group	688	*Escherichia coli*	NRZ-04944
CTX-M-2 group	404	*Escherichia coli*	NRZ-09082
CTX-M-9 group	561	*Escherichia coli*	NRZ 00552
NDM-1	180	*Klebsiella pneumoniae*	JS-37
OXA-51	112	*Acinetobacter baumannii*	DMBF-4

## Results

In total, 684 study participants were included; 54.2% were male, and the median age was 22 years (interquartile range: 14–35 years). More than half of the participants were from rural areas, and 167 (24.4%) could not read or write (Table 3).

**Table 3:** Sociodemographic characteristics of study participants

Sociodemographic characteristics		Numbers	Percentage (%)
Sex	Male	371	54.2
Age	1–15	190	27.8
	16–30	286	41.8
	31–45	110	16.1
	46–60	51	7.5
	> 60	47	6.9
Area of residence	Rural	396	57.9
	Urban	288	42.1
Educational status	Illiterate	167	24.4
	Literate	434	63.5
	Children below school age	83	12.1
Patient settings	Internal medicine ward	129	18.9
	Pediatric ward	131	19.2
	Surgical and Gynecology wards	47	6.9
	Emergency OPD	347	50.7
	ICU	30	4.4

*ICU* intensive care unit, *OPD* outpatient department

### Bacterial cultures

The overall bacterial detection rate across all sample materials was 10.9% (83/761). In 8.7% (n = 66), the isolated bacterium was considered a pathogen, and in 2.2% (n = 17), coagulase-negative staphylococci (CoNS) were isolated and considered clinically irrelevant because of the high likelihood of contamination. The overall blood culture positivity rate was 5.4% (37/684), among which 51.4% (n = 19) were GNB. The overall detection rates in other clinical samples, namely, swabs/pus, urine, and other body fluids, were 60.6% (20/33), 20.8% (5/24), and 37.5% (3/8), respectively. Out of 12 cultured cerebrospinal fluid samples, only one revealed growth of the pathogen *Neisseria meningitidis* (*N. meningitidis*). Overall, 66 pathogenic bacteria were isolated from a total of 761 different clinical samples. Of those, 57.6% (n = 38) were GNB, 39.4% (n = 26) were gram-positive, and 3% (n = 2) were *Candida* species. *Staphylococcus aureus* and *E. coli* were the most prevalent isolates among the gram-positive and gram-negative isolates, respectively. Among the 38 g-negative isolates, 42.1% (n = 16) were *E. coli*, 23.7% (n = 9) were *K. pneumoniae,* and 10.5% (n = 4) were *P. aeruginosa* (for further details see Table 4).

**Table 4:** Frequencies of isolated GNB isolates from different clinical samples from the study participants (n = 38)

Bacterial species	Number	Percentage (%)
*Escherichia coli*	16	42.1
*Klebsiella pneumoniae*	9	23.7
*Pseudomonas aeruginosa*	4	10.5
*Salmonella typhi*	2	5.3
*Enterobacter spp.*	2	5.3
*Acinetobacter baumannii*	1	2.6
*Neisseria meningitidis*	1	2.6
*Raoultella ornithinolytica*	1	2.6
*Raoultella planticola*	1	2.6
*Serratia spp.*	1	2.6

As indicated in Table 5, among all clinical samples, bacterial growth was identified significantly more often among patients with skin and soft tissue infections (SSTIs) (*p* < 0.001). Although not statistically significant, samples from patients with a leukocyte count > 12,000 or < 4000 cells/µL tended to be more likely to reveal bacterial growth (*p* = 0.07). On the other hand, BCs from patients with a diagnosis of acute febrile illness of unknown source were least likely to verify bacteremia compared with other symptoms (*p* = 0.005). Being HIV positive had no statistically significant effect on the blood culture positivity rate (*p* = 0.85). There was no statistically significant difference between the mean value of C-reactive protein (CRP) in positive and negative BCs (mean CRP 71.7 mg/L vs. 64.6 mg/L; *p* = 0.20).

**Table 5:** Culture positivity rate according to clinical diagnosis, source of infection and laboratory parameters

Infectious focus/laboratory parameters	Culture positive (N = 66)	Culture negative (N = 618)	*p* value
	% (n)	% (n)	
Pneumonia/RTI (n = 165)	19.7 (13)	24.6 (152)	0.38
Urinary tract infections (n = 30)	7.6 (5)	4.4 (25)	0.20
Meningitis/encephalitis (n = 57)	6.1 (4)	8.6 (53)	0.48
GITI/hepatitis (n = 43)	6.1 (4)	6.3 (39)	0.94
SSTIs (n = 52)	28.8 (19)	5.3 (33)	** < 0.001**
Acute febrile illness with unknown source (n = 264)	22.7 (15)	40.3 (249)	**0.005**
Sepsis* (n = 32)	4.5 (3)	4.7 (29)	0.96
HIV seropositivity (n = 58)	9.1 (6)	8.4 (52)	0.85
Leukocyte count > 12,000 or < 4000/µL (n = 218)	42.4 (28)	30.7 (190)	**0.07**

	Mean	Mean	
CRP in mg/L	71.7	64.6	0.20

*RTIs* respiratory tract infections, *SSTI* skin and soft tissue infection, *GITI* gastrointestinal tract infection, *CRP* C-reactive protein

*According to the clinician’s diagnosis, regardless of the focus of the infection

Regarding the culture positivity rate among different clinical samples, there was no significant difference between the positivity rates of gram-positive or gram-negative isolates. See Table 6 for details.

**Table 6:** Culture positivity rates among the different clinical samples

Clinical sample	Culture negative	Culture positive	
		Gram-negative isolate % (n)	Other isolates* % (n)
All samples (n = 761)	91.3 (695)	5.0 (38)	3.7 (28)
Blood* (n = 684)	94.6 (647)	2.9 (20)	2.5 (17)
Urine (n = 24)	79.2 (19)	12.5 (3)	8.3 (2)
Swab or pus of infected skin lesion or abscess (n = 33)	33.3 (11)	36.4 (12)	24.2 (8)
CSF (n = 12)	91.7 (11)	8,3 (1)	-
Other body fluids (n = 8)	62.5 (5)	25.0 (2)	(1)

*Gram-positive bacteria (n = 26) and two Candida spp. microbes (yeasts) isolated from blood were considered

### Antimicrobial susceptibility testing

In total, 27 gram-negative isolates were available for susceptibility testing with VITEK® 2. Among those, the resistance rates against commonly used antibiotics at the study site were as follows: ampicillin/sulbactam, 93.3% (n = 21); cefotaxime, 88.9% (n = 24); ceftazidime, 74.1% (n = 20); cefepime, 74.1% (n = 20); ciprofloxacin, 70.4% (n = 19); and gentamicin, 63.0% (n = 17). The resistance rates against rarely used antibiotics were 48.1% (n = 13) for piperacillin/tazobactam and 7.4% (n = 2) for meropenem. Only one case of amikacin resistance was detected among all 27 isolates (Table 7).

**Table 7:** Antibiotic resistance rate of Gram-negative isolates

Bacterial species	Ampicillin	Ampicillin/ sulbactam	Piperacillin	Piperacillin/ tazobactam	Cefotaxime	Ceftazidime	Cefepime	Meropenem	Amikacin	Gentamicin	Ciprofloxacin
*Escherichia coli* (n = 13)	92.3% (12/13)	92.3% (12/13)	92.3% (12/13)	69.2% (9/13)	84.6% (11/13)	84.6% (11/13)	84.6% (11/13)	0% (0/13)	0% (0/13)	53.8% (7/13)	23.1% (3/13)
*Klebsiella pneumoniae* (n = 6)	100% (6/6)	100% (6/6)	100% (6/6)	50.0% (3/6)	100% (6/6)	100% (6/6)	100% (6/6)	16.7% (1/6)	16.7% (1/6)	83.3% (5/6)	50.0% (3/6)
*Pseudomonas aeruginosa* (n = 4)	-	-	50.0% (2/4)	0% (0/4)	100% (4/4)	50.0% (2/4)	50.0% (2/4)	0% (0/4)	0% (0/4)	25.0% (1/4)	75.0% (3/4)
Other (n = 4)	75.0% (3/4)	75.0% (3/4)	75.0% (3/4)	25.0% (1/4)	75.0% (3/4)	25.0% (1/4)	25.0% (1/4)	25.0% (1/4)	0% (0/4)	50.0% (2/4)	75.0% (3/4)
Total (n = 27)	91.3% (21/23)	91.3% (21/23)	85.2% (23/27)	48.1% (13/27)	88.9% (24/27)	74.1% (20/27)	74.1% (20/27)	7.4% (2/27)	3.7% (1/27)	63.0% (17/27)	70.4% (19/27)

Others: *Raoultella ornithinolytica* (n = 1); *Raoultella planticola* (n = 1); *Salmonella* Typhi (n = 1); *Acinetobacter baumannii* (n = 1)

### Resistance genes

The overall frequencies of ESBL and carbapenemase detection among the isolated GNB were 81.5% (22/27) and 7.4% (2/27), respectively. In 55.6% of cases (n = 15), more than one ESBL enzyme was detected in the isolated GNB. The different ESBL enzymes were characterized as TEM (n = 17, 77.3%), CTX-M-1 group (n = 15, 68.2%), SHV group (n = 6, 27.3%) and CTX-M-9 group (n = 2, 9.1%). Both CTX-M-1-type and SHV-type were detected in 5/6 (83.3%) of the *K. pneumoniae* isolates, whereas the SHV group was detected only in 1/13 of the *E. coli* isolates. In *E. coli*, only the CTX-M-1 group was common (see Table 8). Regarding carbapenemases, a single *bla*_NDM-1_ from one isolated *K. pneumoniae* and a combination of *bla*_NDM-1_ plus *bla*_OXA-51_ from an isolate of *A. baumannii* were detected.

**Table 8:** Characterization and frequency of detected ESBL and carbapenemase enzymes among the Gram-negative isolates

Bacterial species	Resistance genes % (n)							
	ESBL enzymes					CP enzymes		
	Any ESBL	CTX-M-1 group	TEM-group	SHV-group	CTX-M-9 group	Any CP	NDM-1	OXA-51
*E. coli* (n = 13)	92.3 (12)	69.2 (9)	76.9 (10)	7.7 (1)	0	0	0	0
*K. pneumoniae* (n = 6)	100 (6)	83.3 (5)	50.0 (3)	83.3 (5)	0	16.7 (1)	16.7 (1)	0
*P. aeruginosa* (n = 4)	50.0 (2)	25.0 (1)	50.0 (2)	0	0	0	0	0
Other* (n = 4)	75.0 (3)	0	50.0 (2)	0	50.0 (2)	25.0 (1)	25.0 (1)	25.0 (1)
All GNB (n = 27)	81.5 (22)	55.6 (15)	63.0 (17)	22.2 (6)	7.4 (2)	7.4 (2)	7.4 (2)	3.7 (1)

*CP* carbapenemase

*Other isolates*: R. planticola* (n = 1) *and R. ornithinolytica* (n = 1): both positive for CTX-M-9 group; *Salmonella* Typhi *(*n = 1): no ESBL- or CPE-production; *and A. baumannii* (n = 1): positive for OXA-51 and NDM-1

### Effectiveness of empiric antibiotic treatment

The results of the locally performed Kirby–Bauer disc diffusion test were available from 25 study participants with *E. coli* and *K. pneumoniae* isolates. Eighteen of these 25 study participants received empirically initiated antibiotic treatment at the time of sampling. The Kirby–Bauer AST revealed high levels of resistance against commonly used antibiotics, rendering 72.2% (13/18) of the initiated antibiotic treatments likely to be ineffective. In particular, 72.0% (18/25) of the isolated GNB were resistant to 3GC, 60.0% (15/25) to fluoroquinolones and 48.0% (12/25) to gentamicin. The results of the initial clinical evaluation, empiric antibiotic treatment and AST results according to the Kirby–Bauer disc diffusion test are summarized below (Table 9).

**Table 9:** Clinical-evaluation, Kirby-Bauer AST result and empirical treatment of participants with *E. coli* or *K. pneumoniae*

Participant No	Bacterial isolate	Resistance against			Clinical diagnosis	Empirical antibiotic treatment	Effectiveness of antibiotic treatment
		3GC	Fluoroquinolones	Gentamicin			
20	*E. coli*	Yes	Yes	No	UTI	Ciprofloxacin	Ineffective*
56	*E. coli*	Yes	No	Yes	RTI	Ceftriaxone, cloxacillin	Effective
258	*E. coli*	No	Yes	Yes	AFI	None	–
259	*E. coli*	Yes	Yes	Yes	AFI	None	–
264	*E. coli*	Yes	No	No	SSTI	Ceftriaxone, metronidazole	Ineffective
272	*E. coli*	Yes	Yes	Yes	SSTI	Cloxacillin	Ineffective
275	*E. coli*	Yes	No	No	SSTI	Ceftriaxone, metronidazole	Ineffective
351	*E. coli*	Yes	Yes	Yes	SSTI	Ceftriaxone metronidazole	Ineffective
411	*E. coli*	Yes	Yes	Yes	AFI	None	–
423	*E. coli*	No	Yes	Yes	RTI	None	–
432	*E. coli*	Yes	Yes	Yes	RTI	Azithromycin, ceftriaxone	Ineffective
440	*E. coli*	Yes	Yes	Yes	UTI	Ceftriaxone	Ineffective
483	*E. coli*	Yes	Yes	No	AFI	Azithromycin, ceftriaxone	Ineffective
503	*E. coli*	No	No	No	RTI	None	–
568	*E. coli*	No	No	No	AFI	None	–
639	*E. coli*	Yes	No	No	UTI	None	–
42	*K. pneumoniae*	No	No	No	AFI	Ceftriaxone, gentamicin	Effective
59	*K. pneumoniae*	No	No	No	RTI	Ceftriaxone, metronidazole	Effective
64	*K. pneumoniae*	No	Yes	No	Meningitis / encephalitis	Ceftriaxone	Effective
278	*K. pneumoniae*	Yes	Yes	Yes	SSTI	Ceftriaxone, cloxacillin	Ineffective
314	*K. pneumoniae*	Yes	Yes	Yes	SSTI	Ceftriaxone, vancomycin	Ineffective
332	*K. pneumoniae*	Yes	Yes	Not	AFI, sepsis	Ceftriaxone, gentamicin	Effective
545	*K. pneumoniae*	Yes	Yes	Yes	UTI	Tuberculostatic treatment	Ineffective
677	*K. pneumoniae*	Yes	Not	No	RTI	Ceftazidime, vancomycin	Ineffective
681	*K. pneumoniae*	Yes	Yes	No	RTI	Ceftriaxone, vancomycin	Ineffective

*3GC* 3rd generation cephalosporin, *UTI* urinary tract infection, *RTI* respiratory tract infection, *AFI* acute febrile illness (febrile disease with unknown source), *SSTI* skin and soft tissue infection

*Ineffective, high likelihood of empiric treatment failure based on in vitro AST result

## Discussion

To date, infectious diseases are one of the most common causes of morbidity and mortality in resource-limited settings, such as Ethiopia [1], but the availability of epidemiological data about causative pathogens and the distribution of AMR remains limited. In this study, we found a high rate of MDR in GNB isolated from febrile patients. As previously described in Uganda, MDR GNB is the main cause of sepsis in febrile cancer patients, with more than 50% of sepsis episodes being caused by *E. coli* infections [15]. Similar to these findings and to the findings of Wasihun et al. (2015) and Moges et al. (2021) from northern Ethiopia [16, 17], *E. coli* was the most prevalent isolated GNB in our study.

The culture positivity rate of 5.4% from BCs was low, which might partially be explained by the fact that the causative pathogen was noncultivable in a proportion of patients (hemoparasites, viruses, or noncultivable bacteria). Similar findings were reported in South Africa [18]. In patients with febrile illness with an unknown source of infection in comparison to other clinical diagnoses, the likelihood of positive BCs was lowest. The highest yield of bacterial cultures was reported from swabs in patients with SSTIs. There was a tendency toward an association between a decreased or elevated leucocyte count or an increased CRP level and blood culture positivity. However, these associations were not significant, and the predictive value of these parameters to guide blood culture diagnostics is insufficient. Thus, our finding confirms a previous report from Italy that CRP level alone is not sufficient to predict blood culture positivity [19]. In this time of widespread AMR and the associated risk of failing antibiotic therapies, blood culture diagnostics are essential to guide the management of bacterial infections. If the broad application of blood culture diagnostics is not possible due to resource limitations, the application of other parameters, such as procalcitonin or monocyte distribution width (MDW), could help to guide the rational use of BCs [20, 21].

In our study, the resistance rates of isolated GNB against commonly used antibiotics were high, severely confining the effectiveness of aminopenicillins in combination with beta-lactamase inhibitors or 3GC for empiric treatment of infections possibly caused by GNB. The lowest resistance rates were found for the meropenem and amikacin. These results are consistent with the results of other recently published data from different parts of Ethiopia, in particular from Addis Ababa [11, 22], Jimma [23] and Bahir Dar [10]. The different resistance rates to certain antibiotics reflect the frequency of antibiotic prescriptions. While aminopenicillins and 3GCs are applied very frequently, carbapenems are hardly used due to their high cost and limited availability. Of note, while the resistance rate for amikacin was very low, many of the isolated GNB were resistant to gentamicin. This difference might be explained by the frequent application of gentamicin at the study center. In contrast, amikacin is almost never applied [24, 25].

Our data revealed overall frequencies of ESBL and carbapenemase production of 81.5% and 7.4% among the isolated GNB, respectively. Of note, all isolates of *K. pneumoniae* were ESBL-positive. These findings are consistent with recently published data from other parts of Ethiopia, where *K. pneumoniae* has also been shown to be the most common ESBL-expressing pathogen, followed by *E. coli* [10, 11, 22].

Regarding the characterization of ESBL enzymes among the gram-negative isolates in our study, TEM-type and CTX-M-1-type were most common, followed by SHV-type and, least frequently, CTX-M-9-type. CTX-M-2 and CTX-M-8/25 were not detected at all. Compared to TEM-type and SHV-type enzymes, CTX-M-type enzymes are more widely disseminated worldwide, and many variants associated with clinically relevant functional heterogeneity have been described. Thus, coexpression of different ESBL types is more common among GNB, which harbors CTX-M-type enzymes [26].

Both the CTX-M-1 group and SHV group were abundantly detected in *K. pneumoniae*, whereas in *E. coli,* the SHV group was detected in only 8% of the isolates (Table 8). This finding matches the report by Ogutu et al. that SHV-type is the predominant ESBL enzyme in *K. pneumoniae* and TEM-type is the most prevalent in *E. coli* [27].

A single *bla*_NDM-1_ carbapenemase gene in a *K. pneumoniae* isolate and *bla*_NDM-1_ plus *bla*_OXA-51_ carbapenemase genes in an *A. baumannii isolate* were detected. The expression of *bla*_NDM-1_ in an *A. baumannii* isolate has previously been reported from the southwestern part of Ethiopia [28], but to our knowledge, no case of *bla*_NDM-1_ presence in *K. pneumoniae* has been reported to date. In general, this finding is not surprising since the *bla*_NDM-1_ carbapenemase genes in *K. pneumoniae* and *A. baumannii* have been commonly reported from other eastern African countries, such as Kenya, Uganda [29], Egypt [30] and Sudan [31].

In this study, most of the antibiotics initiated by the treating physicians for empiric treatment in the participating patients were ineffective according to the AST. However, these data have to be interpreted with caution since most microbiological culturing was performed after initiation of empiric antibiotic therapy, and evaluation of the clinical success rate of the empirically initiated antibiotic therapies was not part of this investigation. Nevertheless, antibiotic resistance impairing the success of empiric antibiotic therapies seems alarmingly common, and local epidemiological resistance data should be taken into account before the initiation of antibiotic therapy [32]. An adaptation of the local empiric antibiotic therapy strategy could be all the more necessary, as the excessive use of 3GC could be one of the main reasons for the spread of ESBL-producing bacteria [33].

Selectively utilizing antibiotics based on the AST result is not only favorable for optimal treatment success but also plays a major role in combating the spread of MDR bacteria. Adequate microbiological culturing before initiation of empiric antibiotic treatment is necessary to enable AST-based antibiotic therapy. As resources are limited and the supply of laboratory materials is unreliable at the study site, as at many other sites in low-income countries, it may not always be possible to perform comprehensive microbiological testing. In such cases, at least surveillance studies with the subsequent establishment of resistance statistics should be carried out to enable calculated antibiotic therapies adapted to the local resistance status. This might help to reduce the imprudent use of antibiotics [8].

A limitation of this study might be the impaired sensitivity of BCs, since for the first 200 participants, a single locally prepared blood culture bottle was inoculated, and subsequently, only one set of standard commercially available blood culture bottles was used for blood culturing. The limited availability and high cost of commercially available blood culture bottles prevent the regular use of such products on site. Molecular resistance testing was not possible for all gram-negative isolates due to loss upon storage and transport. Our study did not investigate whether infections caused by ESBL- or carbapenemase-producing GNB were associated with reduced success of antibiotic therapies or increased mortality.

## Conclusion

In this study, conducted in a tertiary hospital in Ethiopia, we isolated GNB from patients with infectious diseases with high rates of MDR, including 3GC resistance, which is the most commonly used drug class for empiric antibiotic treatment. Based on local AST results, empiric treatment initiated in 72.2% of patients was likely ineffective. As a cause of widespread drug resistance, we found a high prevalence of various ESBL enzymes, with TEM- and CTX-M-1-types predominating. More than half of the gram-negative isolates harbored two or more ESBL genes. In addition, carbapenemases were detected in 7.4% of gram-negative isolates, despite the limited availability and infrequent use of carbapenems in the country. These findings underscore the need for regular microbiological testing of appropriate specimens before initiating empiric antibiotic treatment wherever possible. Therefore, there is an urgent need to strengthen AMS, take appropriate measures to regulate antibiotic use and monitor the emergence of resistant bacteria. In this study, we reported a low positivity rate (5.4%) for BCs, indicating the need for a new diagnostic approach such as plasma cell-free DNA sequencing or the use of MDW- or PCT-guided blood culture to improve the positivity rate.

## Data Availability

All relevant data generated or analyzed during this study are included in this article.

## Abbreviations

3GC: 3Rd generation cephalosporin
AST: Antimicrobial susceptibility testing
BC: Blood cultures
CP: Carbapenemase
CTX-M: Cefotaximase-Munich
EDTA: Ethylene diamine tetraacetic acid
ESBL: Extended-spectrum β-lactamase
EUCAST: European Committee on Antimicrobial Susceptibility Testing
GNB: Gram-negative bacteria
IMP: Imipenemase metallo-β-lactamase
KPC: *K.* *pneumoniae* Carbapenemase
MBL: Metallo-beta-lactamase
MDR: Multidrug resistance
MDW: Monocyte distribution width
NDM: New Delhi metallo-β-lactamase
OXA: Oxacillinase
PCR: Polymerase chain reaction
SHV: Sulfhydryl variable
SOPs: Standard operating procedures
TEM: Temoniera
VIM: Verona imipenemase
